# Mutations in *BRCA1*, *BRCA2*, and *PALB2*, and a panel of 50 cancer-associated genes in pancreatic ductal adenocarcinoma

**DOI:** 10.1038/s41598-018-26526-x

**Published:** 2018-05-25

**Authors:** Shoko Takeuchi, Manami Doi, Naoki Ikari, Masakazu Yamamoto, Toru Furukawa

**Affiliations:** 1 Department of Surgery, Institute of Gastroenterology, Tokyo Women’s Medical University, Tokyo, 162-8666 Japan; 2 Institute for Integrated Medical Sciences, Tokyo Women’s Medical University, Tokyo, 162-8666 Japan; 3 Department of Gastrointestinal Surgery, Tokyo Metropolitan Cancer and Infectious Diseases Center Komagome Hospital, Tokyo, 113-8677 Japan; 4 Department of Surgical Pathology, Tokyo Women’s Medical University Hospital, Tokyo, 162-8666 Japan; 5 Department of Histopathology, Tohoku University Graduate School of Medicine, Sendai, 980-8575 Japan

## Abstract

Mutations in genes of the breast cancer susceptibility gene (BRCA) pathway, namely, *BRCA1*, *BRCA2*, and *PALB2*, can provide useful information for the efficacy of platinum-based or poly ADP-ribose polymerase inhibitors chemotherapeutic regimens. Pancreatic ductal adenocarcinoma (PDAC) is an important target for such precision chemotherapies because of its dismal prognosis. We analyzed mutations in the entire coding regions of the BRCA pathway genes, expression of breast cancer 2 (BRCA2), and mutations in hotspots of 50 cancer-associated genes in 42 surgically resected PDACs, and evaluated their associations with clinicopathological features. We identified 13 rare germline mutations in the BRCA pathway genes; 68 somatic mutations in *KRAS*, *TP53*, *SMAD4*, *CDKN2A*, *GNAS*, *SMARCB1*, and *RB1*; and 2 germline variations in *MLH1*. Among them, *BRCA2*^S2148fs^ was known to be pathogenic. *BRCA2*^R18H^ and *BRCA2*^G2044V^ were enriched in tumor tissues. *BRCA2*^K799R^ and *BRCA2*^R2964T^ were novel germline variations. Patients harboring potentially deleterious mutations in the BRCA pathway genes showed significantly better prognosis than those with benign mutations or no mutation. These results indicate that rare germline variations in BRCA pathway genes could be found more frequently than previously anticipated and, more importantly, potentially deleterious mutations of them could be a favorable prognostic factor in patients with resectable PDACs.

## Introduction

In 2016, pancreatic cancer was the fourth leading cause of cancer death in Japan, as was the case in Western countries, and its incidence and mortality are continuously increasing. Pancreatic ductal adenocarcinoma (PDAC), a common type of pancreatic cancer, is one of the most fatal malignancies, with a 5-year overall survival of less than 10% (Cancer Registry and Statistics. Cancer Information Service, National Cancer Center, Japan; https://ganjoho.jp/reg_stat/statistics/stat/index.html), which has remained unchanged despite many decades of research. Although the most effective treatment for PDAC is surgical resection, it is often diagnosed at an advanced stage, and curative resection is not possible. Control of locally advanced tumors or metastases is critical to improve treatment response of advanced PDACs. Therefore, it is vital to understand the complex genotypes of such advanced tumors.

In the last decade, genomic analyses of PDACs have been extensively conducted, and have confirmed that *KRAS*, *CDKN2A*, *TP53*, and *SMAD4* are commonly and somatically mutated genes^1,2^. Moreover, mutations in genes involved in the breast cancer susceptibility gene (BRCA) pathway, namely, *BRCA1*, *BRCA2*, and *PALB2*, have been uncovered in a subset of PDACs. BRCA pathway-mutated PDACs are presumably defective for DNA double-strand break repair, and may be particularly vulnerable to chemotherapies with platinum-based anticancer drugs or poly ADP-ribose polymerase (PARP) inhibitors. Therefore, investigating the molecular epidemiological and clinicopathological characteristics of BRCA pathway-mutated PDACs is important for designing a therapeutic strategy for pancreatic cancer. However, data on BRCA pathway-mutated PDACs continue to be lacking. PDACs with familial predisposition, which is known as familial pancreatic cancer, show BRCA pathway mutations as one of the most common germline mutations^3^. On the other hand, sporadic PDACs harbor BRCA pathway mutations relatively scarcely; the prevalence of mutations in *BRCA2* in sporadic PDACs has been reported as 3.6% to 7%^4,5^. The data on the prevalence of *BRCA1* and *PALB2* mutations are more limited, but these prevalences have been reported at an even lower rate (<3%)^6^. Additionally, data on mutations of common oncogenes and tumor suppressor genes in BRCA pathway-mutated PDACs and their clinicopathological characteristics remain limited and unclear.

In this study, we aimed to analyze mutations in BRCA pathway genes as well as 50 cancer-associated genes concurrently in apparently sporadic surgically resected PDACs to evaluate molecular epidemiological and clinicopathological characteristics in BRCA pathway-mutated PDACs.

## Results

### Mutations in BRCA pathway genes in PDACs

Studied were 42 patients with histopathologically confirmed PDACs that were surgically resected between 2007 and 2014 at the Tokyo Women’s Medical University Hospital whose frozen tissue samples were available. Clinicopathological features of the patients are listed in Supplementary Table S1. Among them, 5 cases were found to have a family history of pancreatic cancer, with 4 cases that met the definition of familial pancreatic cancer, i.e., two first-degree relatives with PDAC^3^. Therefore, this study cohort consisted of 38 sporadic cases and 4 familial cases.

We performed targeted sequencing analyses of all coding exons of *BRCA1*, *BRCA2*, and *PALB2*, as well as sequencing mutational hotspots of 50 cancer-associated genes (Supplementary Table S2) in the 42 PDAC tumors with paired normal tissues using a next-generation sequencing platform. We obtained sequencing data at an average read depth of 1,043 (range 380–2,841) per amplicon. In data analysis, we collected nonsynonymous somatic mutations as well as nonsynonymous germline variations for which frequencies in the Exome Aggregation Consortium (ExAC) database^7^ were less than 1% (Supplementary Table S3).

We identified 13 mutations in BRCA pathway genes in 12 (28.6%) of the 42 PDACs, all of which were rare germline mutations including 11 in *BRCA2*, 1 in *BRCA1*, and 1 in *PALB2* (Tables 1 and S4). Among these 13 mutations, 2 germline *BRCA2* mutations, *BRCA2*^K799R^ and *BRCA2*^R2964T^, seemed to be novel mutations, because these variants were unreported in any public databases, including 1000 Genome, ExAC, dbSNP, and ESP6500. The germline variation *BRCA1*^M1628T^ was found in a patient with a family history of pancreatic cancer (Case 42). The other 4 patients with a family history of pancreatic cancer did not harbor any BRCA pathway mutations. One patient (Case 11) harbored multiple germline mutations: *BRCA2*^K322Q^ and *BRCA2*^P3292L^. We could not determine whether these mutations were biallelic compound mutations or not. We did not find any somatic mutation in the BRCA pathway genes in our cohort.

**Table 1:** Mutations in BRCA pathway genes in pancreatic ductal adenocarcinoma.

Gene	Status	Coding DNA	Protein	dbSNP	Frequency in ExAC	Prediction*	Allele frequency		Case
							Tumor	Normal	
*BRCA1*	germline	c.4883T > C	p.M1628T	rs4986854	0.0015	B; T; B	0.45	0.50	Case 42
*BRCA2*	germline	c.53G > A	p.R18H	rs80358762	0.00004	B; T; D	0.61	0.49	Case 29
*BRCA2*	germline	c.551T > C	p.L184P	rs80358775	—	C; D; D	0.41	0.45	Case 7
*BRCA2*	germline	c.623T > G	p.V208G	rs80358865	0.00006	U; D; D	0.50	0.45	Case 32
*BRCA2*	germline	c.964A > C	p.K322Q	rs11571640	0.00006	C; D; D	0.55	0.56	Case 11
*BRCA2*	germline	c.1744A > C	p.T582P	rs80358457	0.0002	C; D; D	0.46	0.49	Case 6
*BRCA2*	germline	c.2350A > G	p.M784V	rs11571653	0.0003	B; T; B	0.59	N.A.**	Case 19
*BRCA2*	germline	c.2396A > G	p.K799R	—	—	N; T; B	0.50	0.50	Case 28
*BRCA2*	germline	c.6131 G > T	p.G2044V	rs56191579	0.00004	C; T; B	0.72	0.53	Case 2
*BRCA2*	germline	c.6444_6445del	p.S2148fs	rs80359592	—	P; N; N	0.45	0.50	Case 24
*BRCA2*	germline	c.8891G > C	p.R2964T	—	—	N; D; D	0.48	0.47	Case 35
*BRCA2*	germline	c.9875C > T	p.P3292L	rs56121817	0.00007	C; D; PD	0.38	0.51	Case 11
*PALB2*	germline	c.2228A > G	p.Y743C	rs141749524	0.0001	C; T; B	0.50	0.50	Case 3

*Predictions are noted in the order of ClinVar, SIFT and PolyPhen-2, and abbreviations are B, benign; C, conflicting interpretations of pathogenicity; D, damaging; N, no information; P, pathogenic; PD, possibly damaging; T, tolerant; and U, uncertain significance.

**N.A., not available because existence of this variation was confirmed by Sanger sequencing.

We examined predicted functional effects of all the 13 identified mutations in BRCA pathway genes using online prediction programs, namely, PolyPhen-2 (http://genetics.bwh.harvard.edu/pph2/), SIFT (http://sift.jcvi.org/), and ClinVar (https://www.ncbi.nlm.nih.gov/clinvar/). Seven of the 13 mutations were predicted as at least possibly damaging by SIFT and/or Polyphen-2. By ClinVar, 1 mutation was regarded as pathogenic, 7 as being of uncertain significance or conflicting interpretations of pathogenicity, and 3 as benign. The remaining were “no information” in ClinVar (Table 1). The pathogenic mutation was a frameshift mutation, *BRCA2*^S2148fs^. On the other hand, we found that some mutations, namely, *BRCA2*^R18H^ and *BRCA2*^G2044V^, were apparently enriched in tumor cells, which was evidenced by increasing mutant allele frequencies in tumor tissue compared to paired normal tissue (Table 1). This apparent enrichment in tumor tissues suggests that these mutations may be selected for and confer some advantages upon cancer phenotypes, which indicates a likely pathogenicity, even though they were predicted to be benign for the former and conflicting interpretation of pathogenicity for the latter.

### Mutations in hotspots of 50 cancer-associated genes

**Figure 1:**
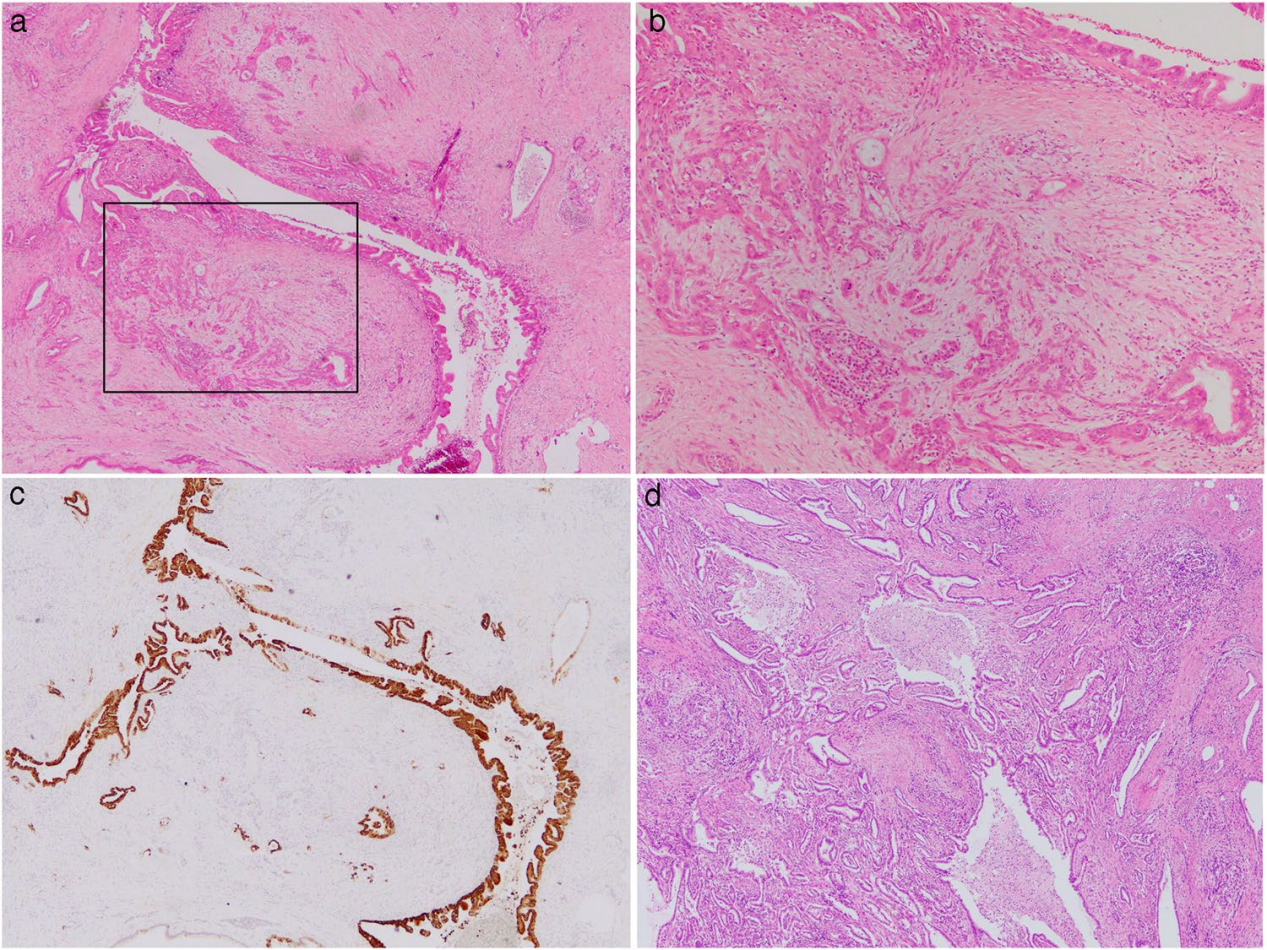
Histopathological images of examined pancreatic ductal adenocarcinomas. The tissue of Case 14 with the somatic mutation of *GNAS* showed cystically dilated ducts with papillary dysplastic cells (**a**) expressing mucin 5AC (**c**) close to solid invading tumors (**b**) the higher magnification image of inset in (**a**)), which indicates that this carcinoma was associated with IPMN. The tissue of Case 30 with the germline mutation of *MLH1* showed pathological findings of usual ductal adenocarcinoma with dense stromal fibrosis (**d**). (**a**,**b** and **d**), hematoxylin and eosin staining, and (c) indirect immunohistochemical staining. Original magnification, ×40 (**a**) ×100 (**b**) ×40 (**c**) and ×40 (**d**).

In analyses of hotspots of 50 cancer-associated genes, we identified somatic mutations in 32 hotspots and a germline mutation in 1 hotspot. In the examined 42 PDACs, 37 (88.1%) harbored somatic mutations in *KRAS*; 14 (33.3%) in *TP53*; 7 (16.7%) in *SMAD4*; 6 (14.3%) in *GNAS*; 2 (4.8%) in *CDKN2A*; 1 (2.4%) in *SMARCB1;* and 1 (2.4%) in *RB1* (Tables 2 and S4). These results indicate that mutations in *KRAS*, *TP53*, *SMAD4*, and *CDKN2A*, the four well-known genes frequently mutated in PDACs, were also prevalent in the current cohort at a rate almost coinciding with published reports^8,9^, although some mutations, i.e., *TP53*^Q104fs^ (c.311_318del), *SMAD4*^H105fs^ (c.316dupA), and *SMAD4*^V409fs^ (c.1227_1228del), seemed to be novel, as they were unreported in the Catalogue of Somatic Mutations in Cancer (COSMIC; http://cancer.sanger.ac.uk/cosmic) database. Mutations in *GNAS* were found in 6 PDACs, which suggested that these PDACs were associated with intraductal papillary mucinous neoplasms (IPMNs), because *GNAS* mutations are known to be exclusive to IPMNs among the diverse pancreatic neoplasms^10–12^. Actually, they contained cystically dilated ducts with papillary dysplastic cells close to solid invading tumors (Fig. 1).

**Table 2:** Somatic and germline mutations in 50 cancer associated genes in pancreatic ductal adenocarcinoma.

Gene	Status	Mutation	Coding DNA	Protein	COSMIC	ClinVar	N*
*KRAS*	somatic	missense	c.35G > A	p.G12D	COSM521	pathogenic	16
*KRAS*	somatic	missense	c.35G > T	p.G12V	COSM520	pathogenic	10
*KRAS*	somatic	missense	c.34G > C	p.G12R	COSM518	pathogenic	7
*KRAS*	somatic	missense	c.34G > T	p.G12C	COSM516	pathogenic	2
*KRAS*	somatic	missense	c.35G > C	p.G12A	COSM522	pathogenic	1
*KRAS*	somatic	missense	c.182A > G	p.Q61R	COSM1158660	pathogenic	1
*TP53*	somatic	missense	c.844C > T	p.R282W	COSM3378339	pathogenic	2
*TP53*	somatic	nonsense	c.916C > T	p.R306X	COSM3388168	pathogenic	1
*TP53*	somatic	missense	c.832C > G	p.P278A	COSM3717626	likely pathogenic	1
*TP53*	somatic	missense	c.824G > A	p.C275Y	COSM2744531	likely pathogenic	1
*TP53*	somatic	missense	c.817C > T	p.R273C	COSM3355991	pathogenic	1
*TP53*	somatic	inframe deletion	c.764_766del	p.I255_T256del	COSM1480062	—	1
*TP53*	somatic	missense	c.733G > A	p.G245S	COSM3356965	pathogenic	1
*TP53*	somatic	frameshift deletion	c.723del	p.S241fs	COSM2744618	—	1
*TP53*	somatic	missense	c.659A > G	p.Y220C	COSM99718	pathogenic	1
*TP53*	somatic	missense	c.524G > A	p.R175H	COSM3355994	pathogenic	1
*TP53*	somatic	missense	c.518T > C	p.V173A	COSM1630438	likely pathogenic	1
*TP53*	somatic	missense	c.413C > T	p.A138V	COSM288785	—	1
*TP53*	somatic	frameshift deletion	c.311_318del	p.Q104fs	—	—	1
*SMAD4*	somatic	frameshift insertion	c.316dupA	p.H105fs	—	—	1
*SMAD4*	somatic	missense	c.326T > G	p.L109R	COSM5196465	—	1
*SMAD4*	somatic	missense	c.1051G > T	p.D351Y	COSM1151549	—	1
*SMAD4*	somatic	missense	c.1081C > A	p.R361S	COSM14151	pathogenic	1
*SMAD4*	somatic	frameshift deletion	c.1227_1228del	p.V409fs	—	—	1
*SMAD4*	somatic	nonsense	c.1333C > T	p.R445X	COSM14096	pathogenic	1
*SMAD4*	somatic	frameshift insertion	c.1587dupA	p.L529fs	COSM5945985	pathogenic	1
*CDKN2A*	somatic	nonsense	c.172C > T	p.R58X	COSM1624870	—	1
*CDKN2A*	somatic	nonsense	c.262G > T	p.E88X	COSM12512	likely pathogenic	1
*GNAS*	somatic	missense	c.601C > T	p.R201C	COSM27887	pathogenic	3
*GNAS*	somatic	missense	c.602G > A	p.R201H	COSM94388	pathogenic	3
*SMARCB1*	somatic	missense	c.215C > A	p.T72K	—	—	1
*RB1*	somatic	nonsense	c.958C > T	p.R320X	COSM1152653	pathogenic	1
*MLH1*	germline	missense	c.428T > A	p.V143D	COSM26085	likely pathogenic	2

*N denotes number of cases.

We detected a germline mutation in *MLH1*, *MLH1*^V143D^, in 2 independent patients. This mutation has been reported as a germline variation found in a family with Lynch syndrome^13^. Therefore, we were curious whether these patients in our cohort might fulfill criteria for Lynch syndrome^13^, and indeed found that 1 of the patients had a past history of colon cancer and one first-degree relative with rectal cancer, although the detailed clinical and family information sufficient for assessing Lynch syndrome was not available. We examined the histology of these patients’ PDACs to determine whether they showed the medullary phenotype that is often seen in PDACs with the mismatch repair deficiency^14^; however, the tissues showed a histology of usual ductal adenocarcinoma with dense stromal fibrosis (Fig. 1). These patients did not harbor any BRCA pathway mutations.

### Expression of breast cancer 2 (BRCA2) protein

**Figure 2:**
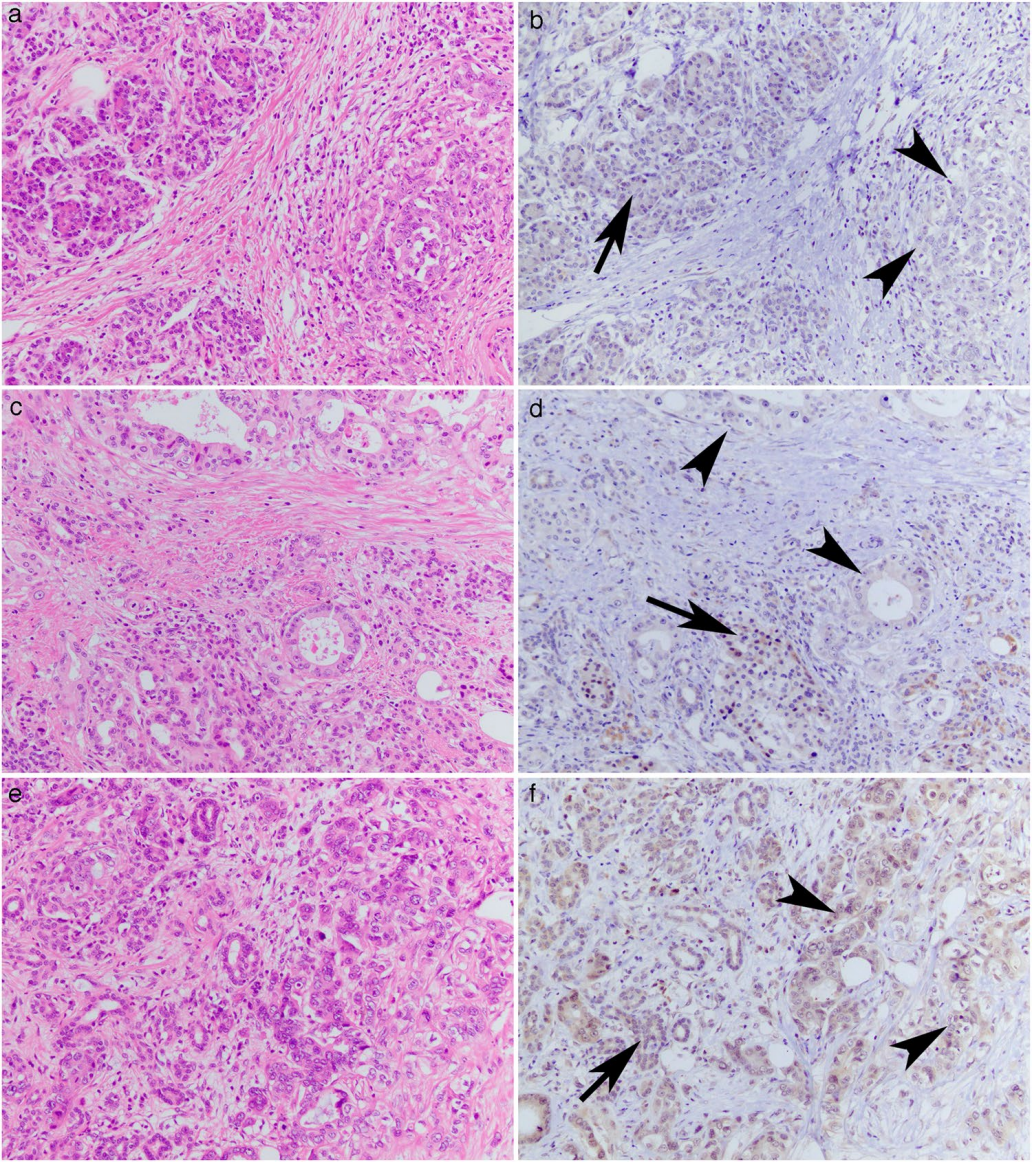
Expression of BRCA2 protein in pancreatic ductal adenocarcinomas. The tissues of Case 17 (**a**,**b**) and Case 19 (**c**,**d**) showed reduced expression of BRCA2 in the ductal carcinomas (arrowheads in b and d) in comparison with adjacent normal acinar and islet cells (arrows in **b** and **d**). The tissue of Case 29 (**e**,**f**), in which the enrichment of a missense mutant allele in the tumor DNA was detected, showed retained expression of BRCA2 in ductal carcinoma (arrowheads in **f**) comparable to the normal acini (an arrow in **f**). Images were hematoxylin and eosin staining (**a**,**c** and **e**) and the indirect immunohistochemical staining with diaminobenzidine as the chromogen (**b**,**d** and **f**). Original magnification, ×200.

Next, we investigated the expression of BRCA2 in the 42 cases by immunohistochemistry. We graded the expression in cancer cells as retained or reduced by comparing their expression with that in adjacent normal ducts, acini, or islet cells. The expression of BRCA2 was observed in both nuclei and cytoplasms, and as reduced in 7 (16.7%) of the 42 analyzed samples (Fig. 2). There was no apparent difference between nuclear staining and cytoplasmic staining in this examined series. When we compared BRCA2 expression and *BRCA2* genotype, we found that 2 of the 7 reduced expression cases harbored mutant alleles, and the remaining 5 cases had wild-type *BRCA2*, in which the reduced expression was not significantly associated with mutation in *BRCA2* (Table 3). The PDAC tissue that harbored the frameshift germline variation, *BRCA2*^S2148fs^, showed retained expression of BRCA2.

**Table 3:** BRCA pathway aberrations and clinicopathological features.

Features	BRCA pathway genes		P	BRCA2 expression		P
	Potentially deleterious mutations*	Wild type or benign mutations**		Retain	Reduced	
*BRCA2* mutation						
Mutant	8	2	—	8	2	1.00
Wild	1	31	—	27	5	
Age at operation						
Mean (range)	68 (53–79)	65 (43–87)	0.40	65 (43–87)	70 (56–77)	0.23
T***						
T1, T2	3	9	0.70	10	2	1.00
T3, T4	6	24		25	5	
N***						
N0	1	12	0.23	11	2	1.00
N1, N2	8	21		24	5	
Stage***						
0	0	0	0.44	0	0	0.52
I	0	1		1	0	
II	0	6		5	1	
III	6	12		13	5	
IVa	3	13		15	1	
IVb	0	1		1	0	
Histology	0	0	0.44			
Tubular adenocarcinoma	8	27	1.00	30	5	0.58
other	1	6		5	2	
Recurrence						
Yes	4	23	0.24	23	4	0.69
No	5	10		12	3	
Previous cancer history						
Yes	1	10	0.40	10	1	0.65
No	8	23		25	6	
Family history of any cancers						
Yes	3	18	0.45	17	4	1.00
No	6	15		18	3	
Family history of pancreatic cancer						
Yes	0	5	0.57	4	1	1.00
No	9	28		31	6	
Prognosis						
5-year overall survival	68.6%	19.2%	0.031	34.3%	0%	0.83

*Patients with mutations predicted as pathogenic, conflicting, uncertain, or no information by ClinVar. **Patients with mutations predicted as benign by Clinvar or those without mutations. ***According to Japan Pancreas Society Classification (6^th^ ed.).

### Association between BRCA pathway mutations and clinicopathological features

**Figure 3:**
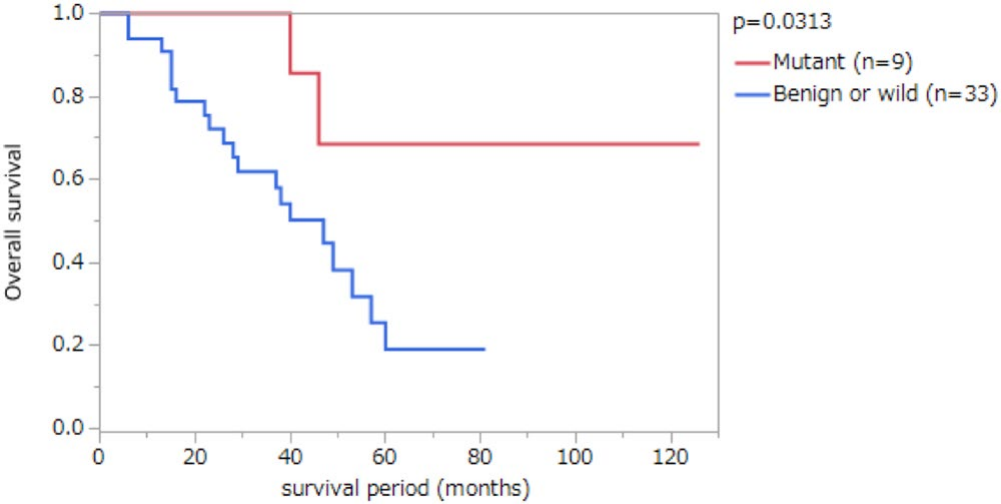
Kaplan-Meier survival analyses of patients with pancreatic ductal adenocarcinomas (PDACs) according to mutations in the BRCA pathway genes. Nine patients with PDACs with potentially deleterious mutations in BRCA pathway genes, namely, *BRCA1*, *BRCA2* and *PALB2* (Mutant), and 33 patients with PDACs with benign mutations or without mutations in the BRCA pathway genes (Benign or wild) were compared. The P value was obtained by Log-rank test.

To know clinicopathological significances of BRCA pathway mutations in PDACs, we divided our cohort into two subcohorts in several ways by their genetic state and compared statistically. We found that patients with potentially deleterious mutations in BRCA pathway genes, i.e., mutations with predictions other than benign by ClinVar including pathogenic, conflicting, uncertain, or no information, showed significantly better prognosis than those without mutations or with benign mutations by ClinVar, in which the 5-year overall survival was 68.6% in the former and 19.2% in the latter (p = 0.031 by logrank test; Fig. 3 and Table 3). This trend was confirmed in a stage-specific manner, i.e., patients with stage III PDAC showed distinct prognosis according to the BRCA pathway genotype (Supplementary Fig. S1). Other clinicopathological features including age, T stage (local tumor invasion), N stage (lymph node metastasis), tumor stage, histology, recurrence, previous cancer history, family history including familial pancreatic cancer were not specifically associated with the BRCA genotypes (Table 3). On the other hand, comparison of prognosis between patients with BRCA mutations including the benign mutations and those without mutation did not show any significant difference. We also found no significant association between BRCA pathway mutations and mutations in *KRAS*, *CDKN2A*, *TP53*, *SMAD4*, or *GNAS* (Supplementary Table S5). In 41 patients with available information in our cohort, 39 patients received adjuvant chemotherapies with gemcitabine, S-1 (tegafur, gimeracil, and oteracil), paclitaxel, cisplatin, and erlotinib. There was no significant difference in administered chemotherapeutic drugs between the patients with potentially deleterious BRCA pathway mutations and those without BRCA pathway mutations or mutations with the benign prediction although cisplatin was administered for 2 patients who had no BRCA pathway mutations in their tumors. We also evaluated the association between expression of BRCA2 and clinicopathological features; however, BRCA2 expression was not significantly associated with any clinicopathological features (Table 3).

## Discussion

In this study, we detected 13 rare germline mutations in BRCA pathway genes in 12 (28.6%) of 42 patients with PDAC including 11 variants of *BRCA2*, 1 of *BRCA1*, and 1 of *PALB2*. Some of these variations seemed to be novel. The prevalence of *BRCA2* germline mutations in our study was one of the highest published, which could be due to (1) different ethnic cohorts because our cohort was consisted of Japanese patients while other published papers used North American cohorts^4,5,15^; and (2) different database for extracting the germline variations, in which we used ExAC^7^, the most reliable exome database currently available, while Grant *et al*. used the 1000 Genome project, dbSNP138, and the National Heart, Lung, and Blood Institute Exome Variant Server ESP6500 data set^4,5,15^. Nevertheless, the results suggest that rare germline variations in BRCA pathway genes may be found in patients with PDAC relatively more frequently than previously anticipated. Hence, testing for mutations in BRCA pathway genes could be warranted not only in familial cases but also in apparently sporadic cases.

The germline variations detected in the BRCA pathway genes could be pathogenic, and 1 frameshift germline variation found in our cohort, *BRCA2*^S2148fs^, was known to be pathogenic by ClinVar. One clue suggesting pathogenicity is the enrichment of a mutant allele in tumor tissues. We observed such an enrichment in 2 missense variations in *BRCA2*, namely, *BRCA2*^R18H^ and *BRCA2*^G2044V^. *BRCA2*^R18H^ is reported in the Kathleen Cuningham Foundation Consortium for Research into Familial Breast Cancer (kConFab) and a Korean breast cancer patients’ cohort as of uncertain variance^16,17^. The R18 residue resides in an interaction domain between BRCA2 and the partner and localizer of BRCA2 encoded by *PALB2*^18^. *BRCA2*^G2044V^ has been found in Japanese, Korean, and Brazilian breast cancer patients^17,19,20^. The Brazilian patient had a family member with hereditary breast and ovarian cancer syndrome^20^. The G2044 residue resides within the BRCA2 repeat domain but outside of the most conserved region^21^. Foci of somatic mutations can also be a clue for pathogenicity. According to the COSMIC database, *BRCA2* somatic mutations have been reported in glioma (*BRCA2*^R18H^), squamous cell carcinoma of the head and neck (*BRCA2*^M784V^), and squamous cell carcinoma of the skin (*BRCA2*^P3292L^).

Notably, the patients with potentially damaging germline mutations of BRCA pathway genes showed better prognosis than those with benign mutations or no mutation. On the other hand, we did not find any significant difference between all patients with BRCA mutations including benign mutations and those without BRCA mutations. This may suggest that deleterious mutations may cause some advantageous effects in prognosis of patients with pancreatic cancer. Deleterious mutations of BRCA pathway genes can induce genomic instability and be a surrogate marker for responsiveness for platinum-based chemotherapy or PARP inhibitor^6^. Golan *et al*. reported that they found no prognostic difference in patients with resectable pancreatic cancer between 25 of those with BRCA mutations and 49 without mutations despite with a trend to increase disease free survival among the BRCA mutation-positive cases treated with neoadjuvant/adjuvant platinum-containing regimens^22^. Some reports have indicated a better prognosis and response to platinum-based treatment in pancreatic cancer patients with BRCA mutations^23,24^. Most of patients of our cohort received an adjuvant chemotherapy based on gemcitabine and S-1 including some with cisplatin, however, there was no significant difference in administration of chemotherapy between the subcohorts, which indicated that the better survival of the subcohort of patients with potentially deleterious mutations was not associated with a chempotherapeutic difference. Hence we could not interpret exactly why the subcohort of patients showed better survivals. Nevertheless, our results may warrant a further study to know prognostic value of BRCA pathway mutations in patients with apparently sporadic pancreatic cancer in a larger cohort.

Familial pancreatic cancer is known to be associated with germline mutations in BRCA pathway genes^25,26^. Our cohort included 4 patients with familial pancreatic cancer, which is consistent with previously published results showing that 7–10% of PDACs correspond to familial pancreatic cancer^3,25^. One of these 4 patients harbored a germline mutation in *BRCA1*, *BRCA1*^M1628T^. This variation has been reported in a breast cancer patient with a family history of breast cancer; however, it has not been previously reported in any case of familial pancreatic cancer^25,27,28^. This variation was predicted to be benign by ClinVar.

We did not find any specific associations between BRCA pathway mutations and mutations in commonly mutated genes, namely, *KRAS*, *CDKN2A*, *TP53*, *SMAD4* and *GNAS*. Mutations in these genes were frequently observed in our cohort in frequencies consistent with published results^8,9^. Besides *KRAS and GNAS*, which usually incurs missense substitutions, *CDKN2A*, *TP53*, and *SMAD4* can be targets of structural variation^29^; therefore, if we could detect structural variations that could be associated with BRCA pathway mutations, there may be some specific associations elucidated, which is an area of further research.

There are some limitations to our study. This study was a retrospective study. The examined cohort was small and included only surgically resected cases. Broader sequencing analysis, such as whole-exome sequencing, may be needed to uncover molecular characteristics associated with BRCA pathway mutations in PDAC.

## Methods

### Subjects and materials

Studied were 42 patients with histopathologically confirmed PDACs surgically resected between 2007 and 2014 at the Tokyo Women’s Medical University Hospital whose frozen tissue samples were available. In all cases, we used frozen tissue samples of tumor and normal tissues (pancreas, spleen, or duodenum) obtained during surgery. We excluded patients who underwent neoadjuvant chemotherapy, because DNA extracted from a treated tumor could be modified by anticancer agents.

This study was approved by the Ethical Committee for genetic studies of the Tokyo Women’s Medical University (protocol #212). The patients analyzed gave relevant informed consent. All research was performed in accordance with relevant guidelines and regulations.

### Tissue dissection and DNA extraction

Methanol-fixed, toluidine-blue-stained sections were prepared from the frozen tissue samples. Tumor and normal tissues were manually dissected and collected separately from the sections under microscopic guidance. Genomic DNA was extracted using a GenElute^TM^ Mammalian Genomic DNA Miniprep Kit (Sigma-Aldrich Corp., St. Louis, MO, USA) according to the manufacturer’s instructions.

### Targeted sequencing analyses by next-generation sequencing

We performed targeted sequencing analyses using the Ion Torrent system (Thermo Fisher Scientific Inc., Waltham, MA, USA). The Ion Ampliseq^TM^ Custom DNA panel (Thermo Fisher Scientific) was used for targeted sequencing to detect mutations in the entire coding regions of *BRCA1*, *BRCA2*, and *PALB2*, and the Ion Ampliseq^TM^ Cancer Hotspot Panel v2 (Thermo Fisher Scientific) was used for targeted sequencing to examine hotspot regions of 50 oncogenes and tumor suppressor genes, as listed in Supplementary Table S2. In both sequencing analyses, the libraries were prepared using the Ion Ampliseq^TM^ Library Kit 2.0 and Ion Xpress^TM^ Library Barcode Adaptors (Thermo Fisher Scientific). The constructed libraries were treated with Ion One Touch^TM^ 2 (Thermo Fisher Scientific) and sequenced using Ion PGM^TM^ sequencer (Thermo Fisher Scientific). Sequencing data were processed with Torrent Suit software (Thermo Fisher Scientific). All procedures were performed according to the manufacturer’s instructions.

### Sanger sequencing

We validated the targeted sequencing data by Sanger sequencing. DNA was amplified by polymerase chain reaction (PCR) using primers listed in Supplementary Table S6 and the Accuprime PCR system (Thermo Fisher Scientific). Amplified products were processed with ExoSAP-IT (GE Healthcare, Buckinghamshire, UK) and sequenced using Bigdye Terminator and a 3130xl Genetic Analyzer (Thermo Fisher Scientific).

### Immunohistochemistry

We evaluated the expression of BRCA2 in tumor and normal tissues by indirect immunohistochemical staining of paraffin-embedded tissues as described previously^30^. The primary antibody used was rabbit polyclonal anti-BRCA2 (CA1033, EMD Millipore Corp., Billerica, MA, USA) produced by using a carboxyl-terminal region (amino acids 3245–3418) of human BRCA2 as an immunogen. Antigen retrieval and dilution of antibody were performed according to the manufacturers’ instructions. Immunohistochemistry of mucin 5AC was performed using mouse monoclonal anti-MUC5AC antibody (NCL-MUC-5AC, Leica Biosystems, Mussloch, Germany) and Ventana BenchMark XT (Ventana Medical Systems, Inc., Tucson, AZ, USA) according to the manufacturers’ instructions.

### Statistics

Clinicopathological and molecular descriptive statistics were calculated for study variables stratified by BRCA pathway mutations (potentially deleterious mutations *vs*. wild type or benign mutations) and BRCA2 expression (retain *vs*. reduced). We used the Fisher’s exact test to compare the distribution of clinicopathological and molecular data because of the small sample sizes. Kaplan-Meier analysis with log-rank test was used to analyze survival. *P* values < 0.05 were considered statistically significant. All statistical analyses were performed using JMP 13 software (SAS Institute Inc., Cary, NC, USA).

### Data availability

The data that support the findings of this study are included in this published article and its supplementary online information files.

## Electronic supplementary material

Supplementary Information
