# Supplementary material for: Mutations in BRCA1, BRCA2, and PALB2, and a panel of 50 cancer-associated genes in pancreatic ductal adenocarcinoma
Source: Sci Rep. 2018 May 25;8:8105. doi: 10.1038/s41598-018-26526-x (PMC5970161; doi:10.1038/s41598-018-26526-x)
Supplement: Supplementary file 1 — Supplementary Information [file 41598_2018_26526_MOESM1_ESM.pdf]

Supplementary Information for

“Mutations in *BRCA1, BRCA2,* and *PALB2,* and a panel of 50 cancer-associated genes in pancreatic ductal adenocarcinoma”

by

Shoko Takeuchi, Manami Doi, Naoki Ikari , Masakazu Yamamoto, and Toru Furukawa

Supplementary Table S1. Clinicopathological features of examined 42 patients

Feature		n (%)*
Age at operation	Mean, range	66 years, 43-87 years
Sex	Male	21 (50.0)
	Female	21 (50.0)
Histology	tubular adenocarcinoma	34 (81.0)
	poorly differentiated adenocarcinoma	5 (11.9)
	mucinous carcinoma	2 (4.8)
	adenosquamous carcinoma	1 (2.4)
pStage	0	0 (0.0)
	I	1 (2.4)
	II	6 (14.3)
	III	18 (42.9)
	IVa	16 (38.1)
	IVb	1 (2.4)
Recurrence	Recurred	27 (64.3)
	Not recurred	15 (35.7)
Previous cancer history	>1 Primary cancer	11 (26.2)
	Breast	3 (7.1)
	Colorectal	3(7.1)
	Ovarian	1 (2.4)
	Other	6 (14.3)
Family history		
FDR and/or SDR with any cancers	≥3	2 (4.8)
	2	6 (14.3)
	1	12 (28.6)
	0	22 (52.4)
FDR and/or SDR with pancreatic cancer	≥3	0 (0.0)
	2	0 (0.0)
	1	5 (11.9)
	0	37 (88.1)
Prognosis	5-year overall survival	0.316

*Number of cases except for age.

FDR, first degree relatives; SDR, second degree relatives

Supplementary Table S2. 50 target genes in the Ion AmpliSeq Cancer Hotspot Panel v2 (Thermo Fisher Scientific)

ABL1	*IDH2*
AKT1	*JAK2*
ALK	*JAK3*
APC	*KDR*
ATM	*KIT*
BRAF	*KRAS*
CDH1	*MET*
CDKN2A	*MLH1*
CSF1R	*MPL*
CTNNB1	*NOTCH1*
EGFR	*NPM1*
ERBB2	*NRAS*
ERBB4	*PDGFRA*
EZH2	*PIK3CA*
FBXW7	*PTEN*
FGFR1	*PTPN11*
FGFR2	*RB1*
FGFR3	*RET*
FLT3	*SMAD4*
GNA11	*SMARCB1*
GNAQ	*SMO*
GNAS	*SRC*
HNF1A	*STK11*
HRAS	*TP53*
IDH1	*VHL*

Supplementary Table S3. Processing of variants obtained by next-generation targeted sequencing

**Germline mutation**
nonsynonymous, GQ≥100, AF≥0.10, ExAC≥0.01 or not reported, validation by Sanger sequencing
**Somatic mutation**
In tumor only, nonsynonymous, GQ≥100, AF≥0.04 in CHPv2: 0.10 in BRCA pathway genes, validation by Sanger sequenicing

AF, allele frequency; GQ, genotype quality; ExAC, Exome Aggregation Consortium;

CHPv2, the Ion AmpliSeq Cancer Hotspot Panel v2 (Thermo Fisher Scientific)

Supplementary Table S4. Mutations identified in BRCA-pathway genes and hotspots of 50 cancer associated genes

Case*	pStage	BRCA-pathway mutations			Hotspot mutations in 50 cancer associated genes			
		Gene	Status	Mutation	Gene	Status	Mutation	AF
1	III	-	-	-	*KRAS*	somatic	G12D	0.33
		-	-	-	*TP53*	somatic	R282W	0.39
		-	-	-	*GNAS*	somatic	R201C	0.26
2	III	*BRCA2*	germline	G2044V	*KRAS*	somatic	Q61R	0.34
					GNAS	somatic	R201H	0.37
3	III	*PALB2*	germline	Y743C	*SMARCB1*	somatic	T72K	0.10
4	III	-	-	-	*KRAS*	somatic	G12R	0.19
5	II	-	-	-	*KRAS*	somatic	G12V	0.07
6	III	*BRCA2*	germline	T582P	*KRAS*	somatic	G12D	0.12
		-	-	-	*GNAS*	somatic	R201H	0.12
7	III	*BRCA2*	germline	L184P	*KRAS*	somatic	G12C	0.32
		-	-	-	*TP53*	somatic	V173A	0.50
		-	-	-	*SMAD4*	somatic	D351Y	0.35
8	IVa	-	-	-	*KRAS*	somatic	G12D	0.08
		-	-	-	*SMAD4*	somatic	L109R	0.06
9	IVa	-	-	-	*KRAS*	somatic	G12R	0.09
		-	-	-	*TP53*	somatic	R175H	0.09
10	IVa	-	-	-	*KRAS*	somatic	G12V	0.08
11	III	*BRCA2*	germline	K322Q	*KRAS*	somatic	G12R	0.09
		BRCA2	germline	P3292L	-	-	-	-
12	IVa	-	-	-	*KRAS*	somatic	G12D	0.21
		-	-	-	*TP53*	somatic	Y220C	0.14
		-	-	-	*RB1*	somatic	R320X	0.21
13	IVa	-	-	-	*KRAS*	somatic	G12D	0.22
14	III	-	-	-	*KRAS*	somatic	G12D	0.30
		-	-	-	*TP53*	somatic	R273C	0.30
		-	-	-	*GNAS*	somatic	R201C	0.23
15	IVa	-	-	-	*KRAS*	somatic	G12V	0.11
		-	-	-	*TP53*	somatic	R282W	0.14
16	III	-	-	-	*KRAS*	somatic	G12C	0.26
17	III	-	-	-	*KRAS*	somatic	G12V	0.18
		-	-	-	*TP53*	somatic	A138V	0.26
18	III	-	-	-	*KRAS*	somatic	G12D	0.04
19	III	*BRCA2*	germline	M784V	*KRAS*	somatic	G12D	0.25
20	III	-	-	-	*KRAS*	somatic	G12D	0.28
21	IVa	-	-	-	*KRAS*	somatic	G12R	0.37
		-	-	-	*TP53*	somatic	R306X	0.09
22	IVa	-	-	-	*KRAS*	somatic	G12V	0.05
		-	-	-	*SMAD4*	somatic	V409fs	0.08
23	II	-	-	-	*KRAS*	somatic	G12V	0.44

24	IVa	*BRCA2*	germline	S2148fs	-	-	-	-
26	IVa	-	-	-	*KRAS*	somatic	G12V	0.21
		-	-	-	*TP53*	somatic	P278A	0.20
27	II	-	-	-	*KRAS*	somatic	G12R	0.24
		-	-	-	*TP53*	somatic	S241fs	0.35
		-	-	-	*MLH1*	germline	V143D	0.47
28	IVa	*BRCA2*	germline	K799R	*KRAS*	somatic	G12D	0.10
29	IVb	*BRCA2*	germline	R18H	*KRAS*	somatic	G12D	0.21
30	IVa	-	-	-	*KRAS*	somatic	G12D	0.20
		-	-	-	*TP53*	somatic	I255_T256del	0.11
		-	-	-	*MLH1*	germline	V143D	0.50
31	III	-	-	-	*KRAS*	somatic	G12D	0.20
		-	-	-	*SMAD4*	somatic	L529fs	0.11
32	III	*BRCA2*	germline	V208G	*KRAS*	somatic	G12V	0.14
		-	-	-	*SMAD4*	somatic	H105fs	0.15
33	III	-	-	-	*KRAS*	somatic	G12R	0.05
		-	-	-	*TP53*	somatic	G245S	0.06
34	II	-	-	-	*KRAS*	somatic	G12V	0.13
		-	-	-	*SMAD4*	somatic	R361S	0.18
35	IVa	*BRCA2*	germline	R2964T	*KRAS*	somatic	G12D	0.05
		-	-	-	*GNAS*	somatic	R201C	0.13
36	IVa	-	-	-	*KRAS*	somatic	G12D	0.39
		-	-	-	*TP53*	somatic	C275Y	0.39
		-	-	-	*CDKN2A*	somatic	E88X	0.37
37	II	-	-	-	*KRAS*	somatic	G12R	0.10
38	I	-	-	-	*KRAS*	somatic	G12A	0.13
		-	-	-	*GNAS*	somatic	R201H	0.11
41	III	-	-	-	*KRAS*	somatic	G12D	0.33
		-	-	-	*SMAD4*	somatic	R445X	0.39
42	II	*BRCA1*	germline	M1628T	*KRAS*	somatic	G12V	0.12
		-	-	-	*TP53*	somatic	Q104fs	0.19
		-	-	-	*CDKN2A*	somatic	R58X	0.14

*No mutations detected in Case 25 at pStage III, Case 39 at pStage Iva, and Case 40 at pStage IVa.

AF, allele frequency.

Supplementary Table S5. BRCA pathway mutations and common hotspots mutations

Genes with common hotspots mutations		BRCA pathway genes		P-value
		Potentially deleterious mutations*	Wild type or benign mutations**	
KRAS	Mutant	7	30	0.29
	Wild	2	3	
TP53	Mutant	1	13	0.23
	Wild	8	20	
SMAD4	Mutant	2	5	0.63
	Wild	7	28	
CDKN2A	Mutant	0	3	1.00
	Wild	9	30	
GNAS	Mutant	3	3	0.10
	Wild	6	30	

*Patients with mutations predicted as pathogenic, conflicting, uncertain, or no information by ClinVar. **Patients with mutations predicted as benign by Clinvar or those without mutations.

Supplementary Table S6. Primers used for PCR and validation by Sanger sequencing

Gene	Exon	Primers	
		Forward	Reverse
MLH1	11	TGCTGGCCCCTCTGGGGAGATGG	TGGCCTGGGGCTGACTGGACAGG
CDKN2A	2	TCCGTCATGCCGGCCCCCAC	GTCCACGGGCAGACGGCCCC
KRAS	2	CATGTTCTAATATAGTCACATTTTC	ATGCATATTAAAACAAGATTTACC
	3	TTCCTACAGGAAGCAAGTAG	CACCTATAATGGTGAATATCTTC
BRCA2	2	AATGTGGCTAGTGGCACCGG	GCAACACTGTGACGTACTGGG
	7	GCATTCTGCCTCATACAGGC	TGACACCACTGGACTACCAC
	10	GACCACATTGGAAAGTCAATGCC	CATTCACAGGCCAAAGACGG
	10	GCTCACAGAAGGAGGACTCC	GCTTCAAACTGGGCTGAACAG
	11	GCTGCAGCATGTCACCCAG	CAGGTGGCAACAGCTCAACG
	11	GAGAAACCCAGAGCACTGTG	GGTGAAGCCTGTTCTTTTCCC
	11	TTGGCTGCAGCATGTCACCC	CAGGTGGCAACAGCTCAACG
	11	CAGTCCCCTTATTCAGTCATTG	CTTCAACATTCTTCAATACTGGC
	11	CGAACATTCAGACCAGCTCAC	GGAAACTTGCTTTCCACTTGC
	22	ACAAGCTCAGATCCAGTTGG	ACAAGCTCAGATCCAGTTGG
	27	GTTTCCACACCTGTCTCAGCCC	CTGAACTGGTGGGAGCAGTCC
RB1	10	GAAATCTGTGCCTCTGTGTG	TACCATGTGCAATACCTGTC
PALB2	5	GCGCCTGATGATAATGACAGGCC	CTGGGCTGCCTGAACTGTCG
BRCA1	9	CACTGCAGGCTTTCCTGTGG	CGGCTAATTGTGCTCACTGTAC
	14	CAGAGGGAACCCCTTACCTGG	GGGGTCAGGCCAGACACC
TP53	4	TGCACCAGCAGCTCCTACAC	CCCAGAATGCAAGAAGCCCAG
	5	CTGTTCACTTGTGCCCTGAC	GTGGAATCAACCCACAGCTG
	5	CGCCATGGCCATCTACAAGC	AACCAGCCCTGTCGTCTCTC
	6	GCGTGTGGAGTATTTGGATGACAG	TGGAGGGCCACTGACAACC
	7	GTGTAACAGTTCCTGCATGGGC	TCAGAGGCAAGCAGAGGCTG
	7	ATCTTGGGCCTGTGTTATCTC	CCAGTGTGATGATGGTGAGG
	8	GCTTCTCTTTTCCTATCCTGAG	GAGATTCTCTTCCTCTGTGC
	8	GCGTGTTTGTGCCTGTCCTG	CCGCTTCTTGTCCTGCTTGC
SMAD4	3	AAACCTGAGCACAGGCCTTG	CCAGGTGATACAACTCGTTCGT
	9	GCTCCTGAGTATTGGTGTTCC	CACCGACAATTAAGATGGAGTG
	10	TGCACATAGGCAAAGGTGTG	CCATTCCTTCCACCCAGATTTC
	11	TGGTGAGCTCCAAGCCACCTTTC	GTTTCCTGCCACGGCTGCTG
	12	GGGAGGATGGGAAGAGATCACCC	AAGGTTAAGGGCCCCAACGG
GNAS	8	ACTGTTTCGGTTGGCTTTGG	GGTAACAGTTGGCTTACTGG
SMARCB1	2	GATACCCCTCACTCTGGAGGCG	CTCCCTGCCCGACTAAGGCC

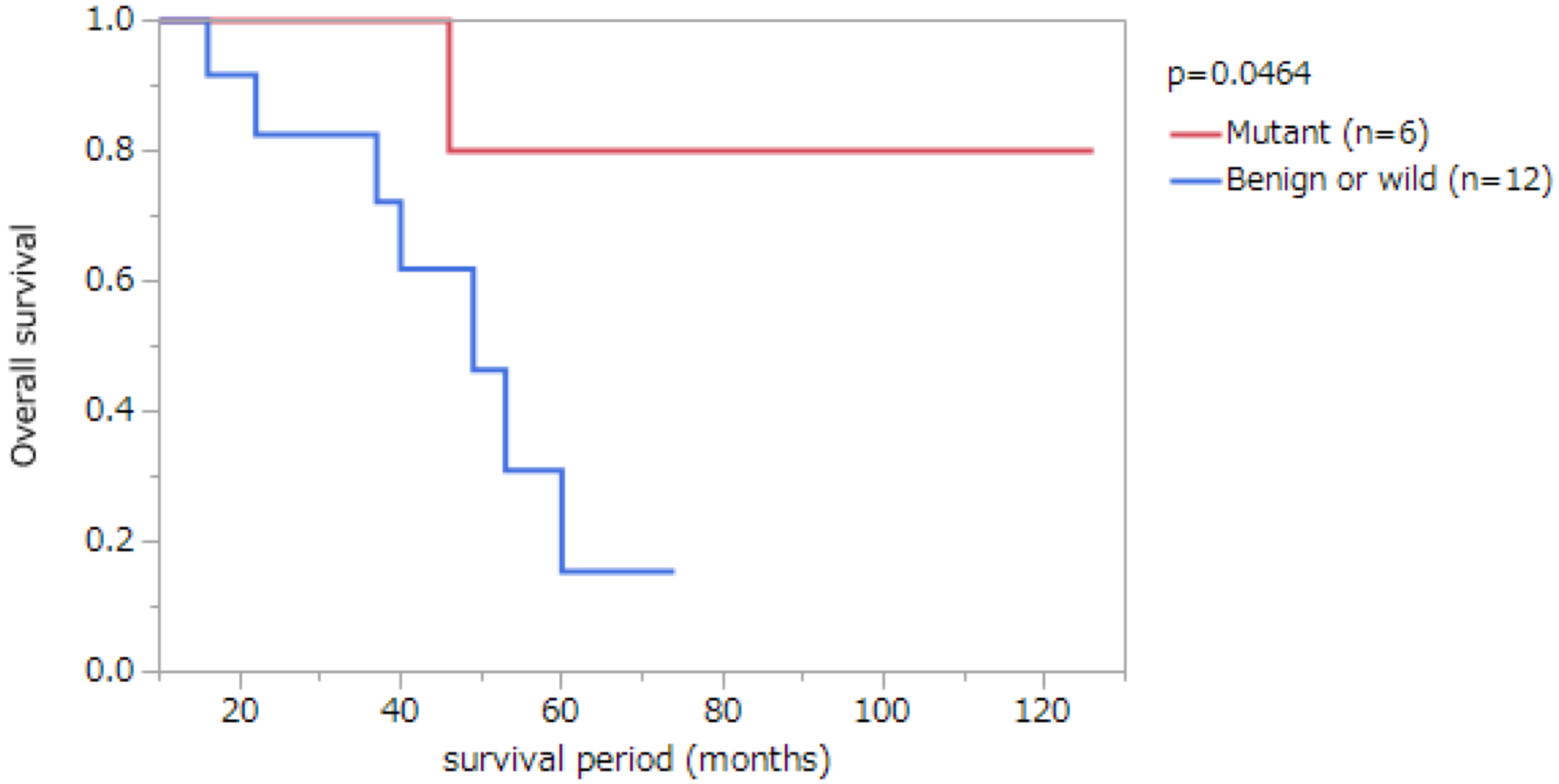

Supplementary Figure S1. Kaplan-Meier survival analyses of patients with pStage III pancreatic ductal adenocarcinomas (PDACs) according to mutations in the BRCA pathway genes. Six patients with PDACs with potentially deleterious mutations in BRCA pathway genes, namely, BRCA1, BRCA2 and PALB2 (Mutant), and 12 patients with PDACs with benign mutations or without mutations in the BRCA pathway genes (Benign or wild) were compared. The P value was obtained by Log-rank test.
